# Increased Frequency of CD4^+^ Follicular Helper T and CD8^+^ Follicular T Cells in Human Lymph Node Biopsies during the Earliest Stages of Rheumatoid Arthritis

**DOI:** 10.3390/cells11071104

**Published:** 2022-03-24

**Authors:** Dornatien Chuo Anang, Tamara H. Ramwadhdoebe, Janine S. Hähnlein, Bo van Kuijk, Noortje Smits, Krijn P. van Lienden, Mario Maas, Daniëlle M. Gerlag, Paul P. Tak, Niek de Vries, Lisa G. M. van Baarsen

**Affiliations:** 1 Amsterdam Rheumatology & Immunology Center (ARC), Department of Rheumatology & Clinical Immunology, 1007 MB Amsterdam, The Netherlands; d.c.anang@amsterdamumc.nl (D.C.A.); ramwadhdoebe@gmail.com (T.H.R.); jhaehnlein@hotmail.com (J.S.H.); b.vankuijk@amsterdamumc.nl (B.v.K.); smits_noortje@hotmail.com (N.S.); dmgerlag@gmail.com (D.M.G.); tak.paulpeter@gmail.com (P.P.T.); n.devries1@amsterdamumc.nl (N.d.V.); 2 Department of Experimental Immunology, Amsterdam Infection & Immunity Institute, Amsterdam UMC, University of Amsterdam, 1007 MB Amsterdam, The Netherlands; 3 Department of Radiology, Amsterdam UMC, University of Amsterdam, 1007 MB Amsterdam, The Netherlands; k.p.vanlienden@amsterdamumc.nl (K.P.v.L.); m.maas@amsterdamumc.nl (M.M.); 4 UCB Pharma, Slough SL1 3XE, UK; 5 Candel Therapeutics, Needham, MA 02494, USA; 6 Department of Internal Medicine, Cambridge University, Cambridge CB2 0QQ, UK

**Keywords:** rheumatoid arthritis, lymph nodes, T lymphocytes, B lymphocytes, follicular T helper cells

## Abstract

Follicular T helper cells (Tfh cells) provide key B-cell help and are essential in germinal center formation and (auto) antibody generation. To gain more insight into their role during the earliest phase of rheumatoid arthritis (RA), we analyzed their frequencies, phenotypes, and cytokine profiles in peripheral blood and lymph node biopsies of healthy controls (HCs), autoantibody-positive individuals at risk for developing RA (RA-risk individuals), and early RA patients. Subsequently, we confirmed their presence in lymph nodes and synovial tissue of RA patients using immunofluorescence microscopy. In the blood, the frequency of Tfh cells did not differ between study groups. In lymphoid and synovial tissues, Tfh cells were localized in B-cell areas, and their frequency correlated with the frequency of CD19^+^ B cells. Compared to lymphoid tissues of healthy controls, those of RA patients and RA-risk individuals showed more CD19^+^ B cells, CD4^+^CXCR5^+^ follicular helper T cells, and CD8^+^CXCR5^+^ follicular T cells. These Tfh cells produced less IL-21 upon ex vivo stimulation. These findings suggest that Tfh cells may present a novel rationale for therapeutic targeting during the preclinical stage of RA to prevent further disease progression.

## 1. Introduction

The presence of autoantibodies years before the presence of clinical signs and symptoms of rheumatoid arthritis (RA) suggests that an increase in B-cell differentiation towards antibody-producing plasma cells occurs very early in the disease [1]. The proliferation and differentiation of B cells are supported by CD4^+^ follicular T helper cells (Tfh cells) [2]. These Tfh cells are derived from naïve CD4^+^ T cells. Following TCR stimulation under the influence of receptor–ligand interactions and the cytokine milieu, T cells may upregulate CXCR5, causing them to migrate to the CXCL13-expressing follicular border, where they interact with B cells and receive signals that will potentially drive them into Tfh cells [3]. Producing cytokines such as IL-21 and IL-4, Tfh cells interact with B cells, which results in B-cell differentiation towards short-lived plasmablasts or in B-cell migration into follicles to contribute to the formation of germinal centers (GCs) [4]. The migration of Tfh cells into B-cell follicles further supports GC formation and drives B-cell differentiation into memory B cells or long-lived antibody-secreting plasma cells. Overall, the net effect of this process depends on the balance between inflammatory and regulatory signals and is tightly regulated to prevent aberrant (auto)immune activation [5].

One of the studies showing the contribution of CD4^+^ Tfh cells in autoimmunity used the sanroque mouse model. Sanroque mice lack Roquin, a repressor of ICOS, resulting in excessive Tfh cell formation and subsequent GC formation. These mice have increased titers of autoantibodies and lupus-like symptoms [6]. In human studies, systemic lupus erythematosus (SLE) patients also show increased frequencies of blood Tfh cells compared with healthy controls (HCs), which correlate with an increase in circulating autoantibodies [7,8,9,10]. Similarly, increased frequencies of peripheral blood Tfh cells have been detected in type I diabetes and RA patients [11,12,13,14,15]. Since Tfh cells can leave lymphoid tissues and relocate through the peripheral blood, they may contribute to B-cell differentiation and the formation of tertiary lymphoid structures at sites of inflammation.

While the role of CD4^+^ Tfh cells in autoimmunity is widely accepted, current data regarding a potential role for CD8^+^CXCR5^+^ follicular-like T cells are less clear and actually point to a dual role. Like CD4^+^ Tfh cells, CD8^+^ Tfh cells express CXCR5, BCL6, IL-21, ICOS, and PD-1 [16,17,18]. An ex vivo study reported that CD8^+^ CXCR5^+^ T cells were able to induce the apoptosis of CD4^+^ Tfh cells, resulting in the inhibition of IL-21 production [19]. However, an antibody-enhancing function of these cells has also been reported in virus-infected mice, where IL-21-producing CXCR5^+^ICOSL^+^CD8^+^ T cells were shown to enhance the production and class-switching of IgG antibodies, revealing a major role of CD8^+^ Tfh cells in the immune response [20].

Studies into the role of T cells in the pathogenesis of RA in humans have mainly focused on the well-known Th1, Th2, Th17, and Treg subsets [21,22,23], during established disease, and in cell populations from peripheral blood and inflamed joints [22,24]. Studies of Tfh cells have focused on peripheral blood samples or inflamed tissue [4,25,26,27], while studies investigating Tfh cells in lymphoid organs during the earliest phases of autoimmunity are lacking. To the best of our knowledge, no study has analyzed CD8^+^ Tfh during the various stages of RA. To gain more insight into the initial activation of Tfh cells in lymphoid tissue and in the role of lymph node Tfh cells during the earliest phases of RA, more research is needed.

In this study, we hypothesized that Tfh cells contribute to autoantibody production in the earliest preclinical phases of RA by driving B-cell differentiation in secondary lymphoid organs. Using human samples acquired by core-needle biopsies of inguinal LNs [28,29], we analyzed and compared the frequencies of CD4^+^ and CD8^+^ Tfh cells in the blood and lymphoid tissue from healthy controls (HCs), autoantibody-positive individuals at risk for developing RA (RA-risk individuals), and early RA patients. In addition, we evaluated the presence of Tfh cells in lymph node germinal centers and inflamed synovial tissue by immunofluorescence microscopy.

## 2. Materials and Methods

### 2.1. Study Subjects

Twenty-four individuals at risk for developing RA (RA-risk) were selected. RA-risk status was defined by the presence of IgM rheumatoid factor (IgM-RF) and/or anticitrullinated protein antibody (ACPA) positivity in subjects without any evidence of arthritis [30]. The median follow-up time was 26.8 months (14.3–39.2 (interquartile range, IQR)), and none of the RA-risk individuals had developed arthritis as yet. We also included 16 early RA patients, based on American College of Rheumatology and European League Against Rheumatism (ACR/EULAR) 2010 criteria [31], who were naïve for disease-modifying antirheumatic drugs (DMARDs) and biologicals, with a disease duration (defined by having arthritis in any joint) of less than 1 year. For comparison, 17 seronegative healthy controls (HCs) were included in the study. The study was performed according to the principles of the Declaration of Helsinki [32] and approved by the institutional review board of the Academic Medical Centre of the University of Amsterdam, and all study subjects provided written informed consent. The demographics of all study subjects are listed in Table 1.

### 2.2. Sample Processing and Cell Culture

Ultrasound-guided inguinal LN biopsies were taken and immediately processed for flow cytometry analysis, snap-frozen en bloc in Tissue-Tek OCT compound (Miles, Elkhart, IN, USA) for immunohistochemistry analysis, or snap-frozen for RNA isolation as described previously [28]. For flow cytometry analyses, LN biopsies were put through a 70 μm cell strainer (BD Falcon, San Jose, CA, USA) to obtain a single-cell suspension. Peripheral blood mononuclear cells (PBMCs) were isolated using standard density gradient centrifugation using Lymphoprep (Nycomed AS, Oslo, Norway) and stored in liquid nitrogen until further use. Freshly isolated LN cells and thawed PBMCs were incubated in RPMI culture medium (Life Technologies, Thermo Fisher Scientific Inc., Waltham, MA, USA) for 4 h in the presence or absence of phorbol myristate acetate (PMA) and ionomycin with brefeldin A (all from Sigma Aldrich, St Louis, MO, USA) and Golgi Stop (BD Biosciences, San Jose, CA, USA). After 4 h, cells were washed and analyzed by flow cytometry. If possible, paired peripheral blood and lymph node samples were collected from RA-risk individuals and RA patients. From healthy controls, either LNs or PBMCs were collected and analyzed. Due to the limited number of cells obtained from some lymph node biopsies, not all samples could be fully analyzed in the study.

Synovial biopsies of the knee joints of RA patients were collected during mini-arthroscopies as described previously [33]. Six to eight samples were snap-frozen together to correct for sampling error [33].

### 2.3. Antibodies and Flow Cytometry Analysis

Cells were stained for 30 min at 4 °C in PBS containing 0.01% NaN_3_ and 0.5% BSA (Sigma Aldrich) with directly labeled antibodies against: CXCR5 Alexa Fluor 488 (clone RF8B2), CCR7 PE-Cy7 (clone 3D12), CD4 APC-H7 (clone SK3), CD8 V450 (RPA-T8), CD3 V500 (UCHT1) (all from BD Biosciences, San Jose, CA, USA), PD-1 PE (J105), CD45 RA (L307.4) eFluor450 (all from eBioscience Inc., San Diego, CA, USA), CD19 V500 (HIB19), and CD45 APC-H7 (2D1). For intracellular cytokine staining, we used IL-10 Pe-Cy7 (clone JES3-9D7) (Biolegend, San Diego, CA, USA) and IL-21 Alexa Fluor 647 (clone 3A3-N2.1) (BD Biosciences). The gating procedure was carefully set up prior to the study. First, all antibodies used were titrated to an optimal concentration. Fluorescence minus one (FMO) and appropriate compensation controls were determined for each antibody in order to gate positive populations. Additionally, an unstained control (for stimulated and unstimulated cells) for each sample was taken during the acquisition (both intracellular and extracellular) to determine positive events.

Cells were analyzed on a FACS Canto II (BD Biosciences), and data were analyzed using FlowJo software (FlowJo, Ashland, OR, USA). Data were plotted as frequency of positive cells.

### 2.4. Immunofluorescent Microscopy

Biopsies were embedded in OCT Tissue TEK and stored in liquid nitrogen. Frozen sections were cut (5 μm) using a cryostat, after which sections were stored at −80 °C until further use. For staining, sections were thawed and air-dried at room temperature and subsequently fixed with acetone [34]. Sections were washed and stained with monoclonal primary antibodies or isotype controls: rat anti-human CXCR5-Alexa Fluor 488 IgG2b (BD Biosciences), rabbit anti-human Bcl-6 IgG (Bio SB, Santa Barbara, CA), mouse anti-human ICOS IgG1 (Novo Biologicals, Littleton, CO, USA), rat IgG2b Alexa Fluor 488 (isotype control, BD Biosciences), mouse IgG1 (isotype control, Dako Cytomation, Heverlee, Belgium), or rabbit IgG (isotype control, Dako Cytomation) diluted in PBS/1% BSA/10% normal human serum (NHS; Lonza, Basel, Switzerland) overnight at 4 °C. After washing with PBS, (directly labeled) secondary antibodies (goat anti-rabbit IgG Alexa 546 and goat anti-mouse IgG1 Alexa 633, both from Life Technologies, Bleiswijk, The Netherlands) were incubated for 30 min in PBS/1%BSA/10% NHS. After washing with PBS, slides were covered with Vectashield containing DAPI (Vector Laboratories, Burlingame, CA, USA) and analyzed on a confocal imaging microscope (Leica Microsystems, Wetzlar, Germany).

### 2.5. Statistical Analysis

Data were tested for normality with the D′Agostino and Pearson omnibus test and are presented as mean with standard deviation or median with IQR. Differences between groups were analyzed using the Kruskal–Wallis test or one-way analysis of variances (ANOVA). Correlations were calculated with Spearman′s rank correlation coefficient for samples that were fully analyzed. All statistical analyses were performed using GraphPad Prism Software (version 6, GraphPad Software, Inc. La Jolla, CA, USA). Results with *p* values < 0.05 were considered statistically significant.

## 3. Results

### 3.1. The Frequency of Peripheral Blood CD4^+^ Circulating Follicular Helper T and Circulating CD8^+^ Follicular T Cells Is Comparable between Healthy Controls, RA-Risk Individuals, and Early RA Patients

**Figure 1:**
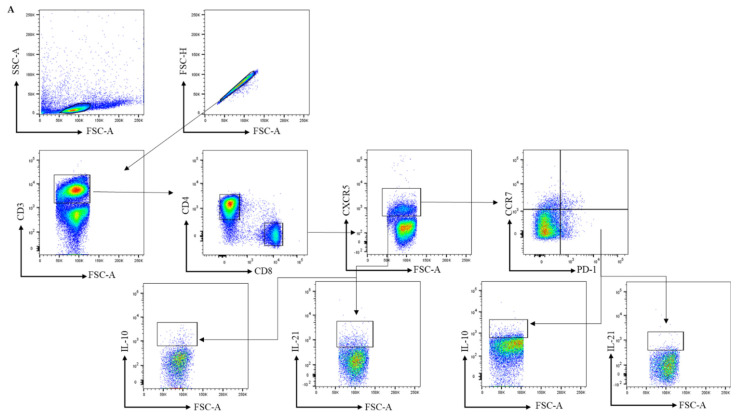

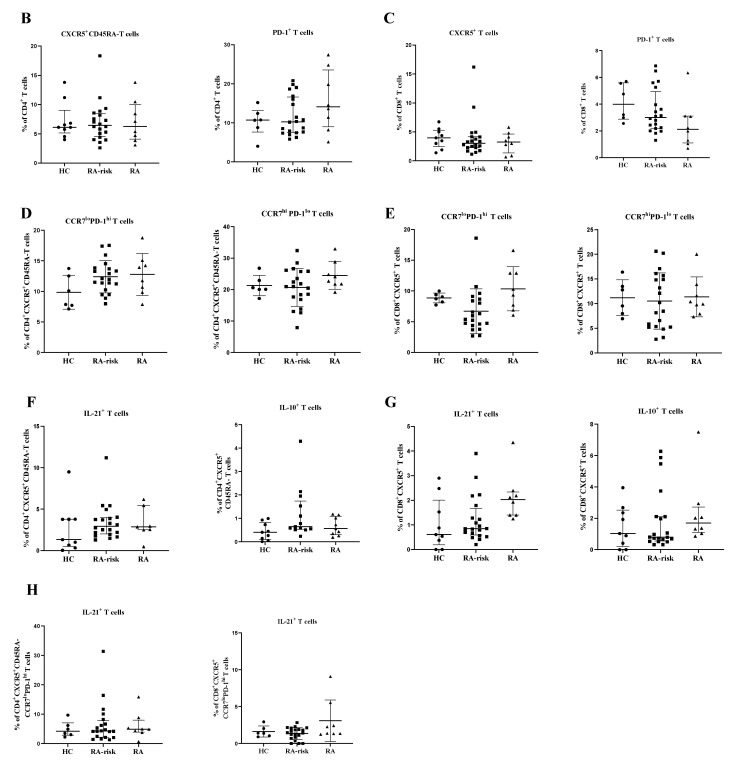
Analysis of circulating follicular helper T cells in peripheral blood samples. (**A**) Gating strategy for cTfh cells in PBMCs using markers for CD3, CD4, CD8, CXCR5, CCR7, PD-1, IL-21, and IL-10. After gating for single cells, CD4^+^ and CD8^+^ T cells were gated within the CD3^+^ population and further characterized. (**B**,**C**) The frequencies of CXCR5^+^ CD45RA-, and PD-1^+^ cells within CD4^+^ and the frequencies of CXCR5^+^ and PD-1^+^ cells within CD8^+^ T cells were analyzed in PBMC samples collected from healthy controls (HC, n = 9 for Figure 1B,C,F,G; otherwise, n = 6), RA-risk individuals (RA-risk, n = 20), and early RA patients (RA, n = 8). (**D**,**E**) The frequencies of blood CCR7 ^low^PD1 ^high^ cTfh and CCR7 ^high^PD1 ^low^ cTfh within the CD4^+^CXCR5^+^CD45RA- and CD8^+^CXCR5^+^ populations are plotted for healthy controls (HC, n = 6), RA-risk individuals (RA-risk, n = 20), and early RA patients (RA, n = 8). (**F**–**H**) The frequencies of IL-21- and IL-10-producing cells within the indicated T-cell subsets are shown for healthy controls (HC, n = 6), RA-risk individuals (RA-risk n = 20), and early RA patients (RA, n = 8). Non-normally distributed data (Figure 1B–E) are presented as median with IQR, and normally distributed data (**F**–**H**) are presented as mean with SD. For statistical analysis, Kruskal–Wallis or one-way ANOVA (when appropriate) was performed. Non-parametric analysis was performed on study groups with n < 8. Shapiro–Wilk test was carried out to further confirm normality in this group. All symbols represent data from single individuals (• healthy controls (HC); ■ RA-risk individuals (RA-risk); ▲ early RA patients (RA)).

To analyze the frequency of circulating peripheral blood CD4^+^ cTfh and circulating CD8^+^ follicular T cells in HCs, RA-risk individuals, and early RA patients, we first analyzed the frequencies of CXCR5^+^ and PD-1^+^ cells within CD4^+^ and CD8^+^ T cells (see gating in Figure 1A and Supplementary Figure S1 (unstained negative control)). We observed a trend towards increased CD4^+^PD-1^+^ T (*p* value < 0.09) in RA patients when compared to healthy controls. However, the frequencies of CD4^+^CXCR5^+^ CD45RA- cells as well as CD8^+^CXCR5^+^ and CD8^+^PD-1^+^ T cells in early RA and RA-risk individuals when compared to healthy controls were on average comparable among the three study groups (Figure 1B,C). Within the CD4^+^CXCR5^+^CD45RA- T-cell subset, CCR7 ^low^PD1 ^high^ cells are described as active Tfh cells, and CCR7 ^high^PD1 ^low^ cells are characterized as quiescent Tfh cells [14]. In our analysis, the frequencies of active (CCR7 ^low^PD-1 ^high^) and quiescent (CCR7 ^high^PD-1 ^low^) cTfh cells within the blood CD4^+^CXCR5^+^CD45RA- and CD8^+^CXCR5^+^ cells were not significantly different among the three study groups, though a trend (*p* = 0.09) was observed for active cTfh (CCR7 ^low^PD-1 ^high^) in the blood of RA patients when compared to healthy controls (Figure 1D,E). No significant correlations between the frequencies of various cTfh cells and age, autoantibodies, or other clinical parameters (TJC, SJC, and ESR) were detected in the blood (data not shown).

Next, we analyzed the capacity for cytokine production in blood cTfh cells upon ex vivo stimulation with PMA/ionomycin. The frequencies of CD4^+^CXCR5^+^CD45RA-IL-21^+^, CD4^+^CXCR5^+^CD45RA-Il-10^+^, CD8^+^CXCR5^+^IL-21^+^, and CD8^+^CXCR5^+^IL-10^+^ T cells were on average comparable among the three study groups (Figure 1F,G). As expected, in the peripheral blood, the frequency of active Tfh cells producing Il-21 was higher than the frequency of quiescent Tfh cells. Finally, the frequency of IL-21^+^ cells among active CD4^+^CXCR5^+^CCR7 ^low^PD-1 ^high^ and CD8^+^CXCR5^+^CCR7 ^low^PD-1 ^high^ was on average comparable among the three study groups (Figure 1H).

Taken together, the frequencies of blood CD4^+^ cTfh and circulating CD8^+^ follicular T cells are highly variable but, on average, not different between RA-risk individuals and early RA patients compared with healthy controls (HCs).

### 3.2. CD4^+^ Follicular Helper T and CD8^+^ Follicular T Cells Are Increased in Lymphoid Tissue of RA Patients

**Figure 2:**
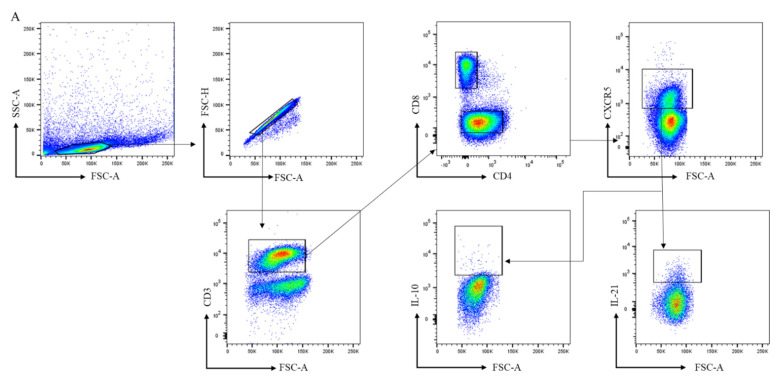

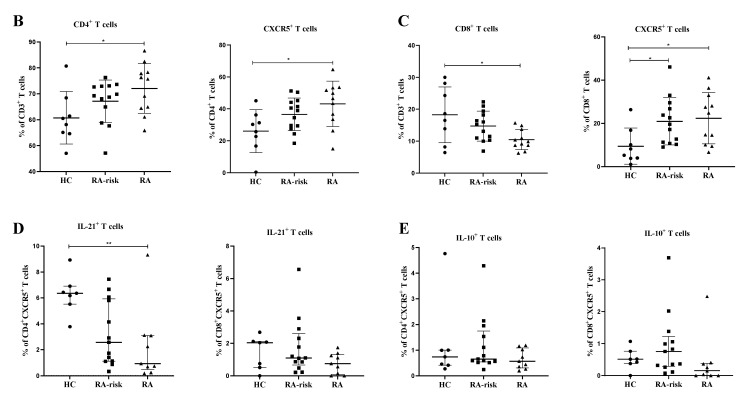
Analysis of follicular helper T cells in lymph node biopsies. (**A**) Gating strategy for follicular-like T cells in lymph node biopsies using markers for CD3, CD4, CD8, CXCR5, IL21, and IL-10. For gating of CD4^+^ cells after stimulation, the largest CD3^+^CD8^-^ population was considered to be CD4^+^ cells, as our unstimulated cells indicated that this corresponds to CD4^+^ cells. This approach was used because the downregulation of CD4 was observed in lymph nodes upon PMA/ionomycin stimulation. (**B**,**C**) Frequencies of CD4^+^, CD4^+^CXCR5^+^, CD8^+^, and CD8^+^CXCR5^+^ T cells in lymph nodes are shown for healthy controls (HC, n = 8), RA-risk individuals (RA-risk, n = 13), and early RA patients (RA, n = 11). (**D**,**E**) The frequencies of IL-21^+^ and IL-10^+^cells within the CD4^+^CXCR5^+^ and CD8^+^CXCR5^+^ populations are plotted for healthy controls (HC, n = 7), RA-risk individuals (RA-risk, n = 13), and early RA patients (RA, n = 9). Non-normally distributed data are presented as median with IQR, and normally distributed data are presented as mean with SD. For statistical analysis, Kruskal–Wallis or one-way ANOVA (when appropriate) was performed, and significant differences are indicated as * *p* < 0.05 or ** *p* < 0.01. Non-parametric analysis was conducted on study groups with n < 8. Shapiro–Wilk test was conducted to further confirm normality in this group. All symbols represent data from single individuals (• healthy controls (HC); ■ RA-risk individuals (RA at-risk); ▲ early RA patients (RA)).

Next, we analyzed the frequencies of CD4^+^ Tfh and CD8^+^ follicular T cells based on CXCR5 expression in LN biopsies (see gating in Figure 2A). CXCR5 expression defines LN T cells that are capable of moving towards the follicular border, where they can interact with B cells [2]. Among CD3^+^ T cells, the frequency of total CD4^+^ T cells was not significantly higher in RA-risk individuals compared to HCs but significantly increased in early RA individuals compared to HCs (Figure 2B). Among CD4^+^ T cells, the frequency of CXCR5^+^ CD45RA- Tfh cells was increased in early RA patients compared with HCs (Figure 2B; *p* < 0.05), while frequencies in RA at-risk individuals were in between those of HC and early RA patients. We next evaluated the frequencies of CD8^+^ T cells. Among CD3^+^ cells, the frequency of CD8^+^ T cells was similar between RA-risk individuals and HCs, while it was significantly lower in early RA patients compared to HCs (Figure 2C). Among CD8^+^ T cells, the frequency of CD8^+^CXCR5^+^ follicular T cells was markedly increased (*p* < 0.05) in RA-risk individuals as well as early RA patients when compared to HCs (Figure 2C).

Since IL-21 and IL-10 production was below the detection limit in unstimulated cells, we analyzed IL-21 and IL-10 production in LN CD4^+^, CD8^+^, CD4^+^CXCR5^+^ Tfh, and CD8^+^CXCR5^+^ follicular T cells upon ex vivo stimulation with PMA/ionomycin. Among CD4^+^CXCR5^+^ Tfh cells, the frequency of IL-21-producing cells upon ex vivo stimulation was significantly decreased in early RA patients (*p* < 0.01) when compared with HCs, while the frequency was intermediate for the RA-risk individuals (Figure 2D). Similar findings were observed for CD8^+^CXCR5^+^ Tfh cells but did not reach statistical significance (Figure 2D). In contrast, the frequency of IL-10 producers in the CD4^+^ Tfh and CD8^+^ CXCR5^+^ follicular T cells was comparable among the three study groups (Figure 2E).

### 3.3. In Lymphoid Tissue, the Frequencies of CD4^+^ Follicular Helper T Cells and CD8^+^ Follicular T Cells Correlate with the Frequencies of CD19^+^ B Cells

**Figure 3:**
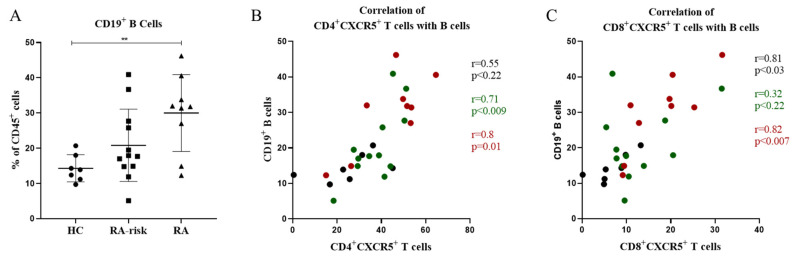
Correlation of CD19^+^ B cells with CD4^+^ follicular helper and CD8^+^ follicular T cells in LNs. (**A**) Frequencies of CD19^+^ B cells in LNs are shown for HCs (n = 7), RA-risk (n = 12), and early RA (n = 9) patients. (**B**) Correlation between the frequencies of CD4^+^CXCR5^+^ Tfh cells and the frequencies of CD19^+^ B cells in LNs. (**C**) Correlation between the frequencies of CD8^+^CXCR5^+^ follicular T cells and the frequencies of CD19^+^ B cells in LNs. Black dots represent correlations in healthy controls, green dots represent correlations in RA-risk individuals, and red dots represent correlations in early RA patients. Error bars represent mean with SD. For statistical analysis, Kruskal–Wallis or one-way ANOVA (when appropriate) was performed, and significant differences are indicated as ** *p* <0.01. All symbols represent data from single individuals (• healthy controls (HC); ■ RA-risk individuals (RA at-risk); ▲ early RA patients (RA)).

Since CD4^+^CXCR5^+^ Tfh and CD8^+^CXCR5^+^ follicular T cells were increased in the LNs of RA patients compared to HCs, we investigated whether they were in any way related to the frequencies of B cells found in LNs. Consistent with previous findings [29], the frequency of CD19^+^ B cells was increased in early RA patients when compared with healthy controls (*p* < 0.05) and showed an elevated trend in RA-risk individuals when compared to healthy controls (Figure 3A). When compared with CD4^+^CXCR5^+^ Tfh cells, we found a strong and significant correlation between the frequencies of CD19^+^ B cells and CD4^+^CXCR5^+^ Tfh cells (*p* < 0.0001, r = 0.76) (Figure 3B). Interestingly, we also observed a significant and strong correlation (*p* < 0.0004, r = 0.62) between CD8^+^CXCR5^+^ follicular T cells and CD19^+^ B cells in LNs (Figure 3C). Taken together, the frequencies of CD4^+^CXCR5^+^ and CD8^+^CXCR5^+^ Tfh cells strongly correlate with the frequencies of CD19^+^ B cells localized within LNs.

### 3.4. In Lymphoid and Synovial Tissue, Follicular T Cells Are Located in B-Cell Areas

**Figure 4:**
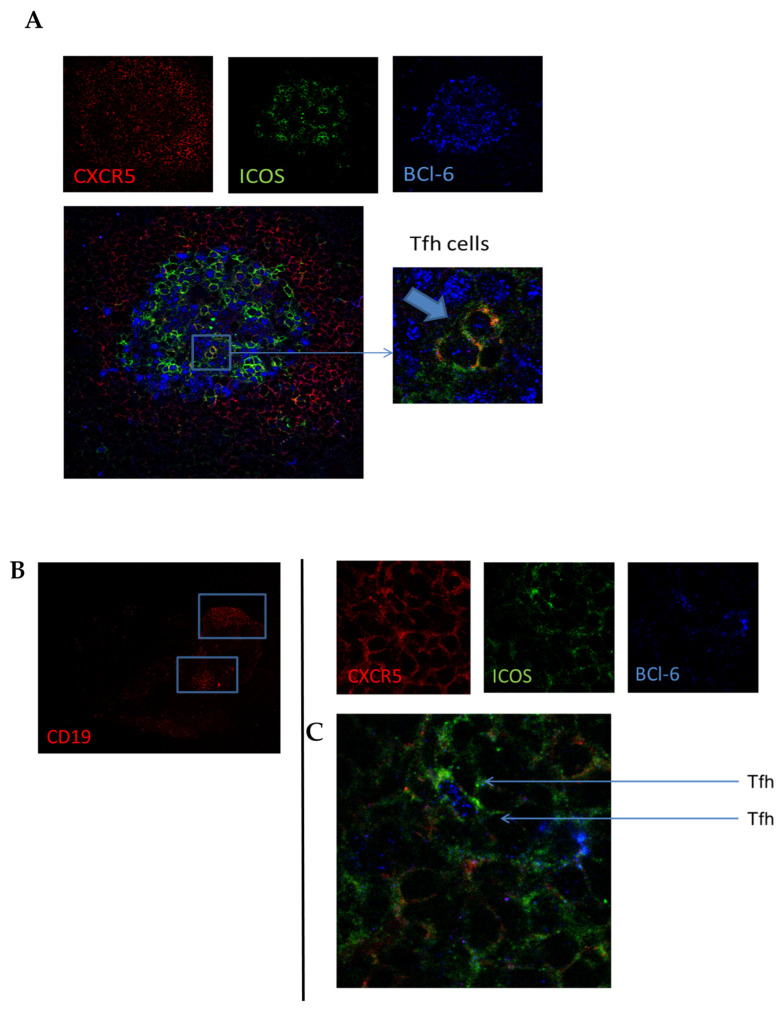
Confirmation of follicular T cells in (**A**) human lymph node tissue. (**B**,**C**) Identification of B cells (**B**) and follicular T cells (**C**) in B-cell-rich areas of consecutive synovial tissue sections. Lymph node tissue sections and synovial tissue sections from RA patients were stained for CXCR5, ICOS, and Bcl6. Pictures are representative of n = 5 experiments. In 63 × zoom, image arrows indicate triple-positive Tfh cells.

To confirm the presence of follicular T cells in lymphoid tissue in vivo, we stained tissue sections from HC and early RA patients for CXCR5 (red), ICOS (green), and Bcl-6 (blue) (Figure 4A). Although CXCR5^+^ICOS^+^Bcl-6^+^ Tfh cells were difficult to find due to their overall low frequency, we were able to identify them in germinal centers present in lymph node biopsies (Figure 4A). Quantification of the number of follicular T cells was not possible due to their limited number and variation in germinal centers present in the analyzed tissue sections. We next investigated whether CXCR5^+^ICOS^+^Bcl-6^+^ Tfh cells were also present in the synovial tissue of RA patients. In B-cell-rich areas of synovial tissue sections (Figure 4B), we could indeed identify these Tfh cells, as shown in Figure 4C. These data show that follicular T cells can be detected with immunofluorescent staining in lymph node biopsies and synovial tissue, which may help identify tertiary lymphoid structures in inflamed tissue.

## 4. Discussion

We studied CD4^+^ Tfh and CD8^+^ follicular T cells in both LN biopsies and peripheral blood samples obtained during the earliest phases of RA and compared our findings to control samples. We found increased frequencies of CD4^+^CXCR5^+^ follicular helper T cells and CD19^+^ B cells in the lymphoid tissue of early RA patients, and CD8^+^CXCR5^+^ follicular T cells were similarly increased in the LN tissue of both RA-risk individuals and RA patients. This increased frequency of B cells in LNs of RA patients compared to healthy controls is in accordance with previous reports [29,35]. A plausible explanation behind this increase could be the retention of B cells in lymphoid tissue, where they eventually differentiate into various B cell effector phenotypes and contribute to disease development. In LN biopsies, the frequencies of CD4^+^CXCR5^+^ and CD8^+^CXCR5^+^ cells correlate significantly with the frequency of CD19^+^ B cells, suggesting an increased number of B and T cells that can possibly interact at the LN follicular border and drive immune responses. In mice, the location of CD4^+^ Tfh cells in LNs during primary and memory responses has been studied in detail [36]. During the primary immune response, CD4^+^ Tfh cells are located in the GC follicle. Indeed, we found Tfh cells in B-cell follicles of lymphoid tissues of RA patients. For a memory response, CD4^+^ Tfh cells are located in the subcapsular region and can leave the follicle via the lymphatic flow. This enables CD4^+^ Tfh cells to migrate through blood towards other secondary lymphoid organs or inflamed tissues, where they can initiate new GC responses if the antigen is present [14]. These migrating CD4^+^ Tfh cells in the blood can be present before differentiation towards a fully mature effector Tfh phenotype [37]. In our study, although not significant, we observed a trend towards an increased frequency of active CD4^+^ Tfh cells in the blood, while quiescent CD4^+^ Tfh cells were not significantly altered in RA and RA-risk individuals when compared with HCs.

CD8^+^ T cells often function as the effector cytotoxic T cell type; hence, they were assumed to be excluded from entry into B-cell follicles and participation in GC reactions [38,39]. However, recent data suggest that CD8^+^ T cells, like their CD4^+^ counterparts, are able to acquire CXCR5, which enables their migration into B-cell follicles, where they subsequently eliminate virus-infected B cells as well as CD4^+^ Tfh cells [19]. Our findings on the presence and increased frequency of CD8^+^ Tfh cells in the lymphoid tissues of early RA patients confirm the presence of CD8^+^ Tfh cells in lymphoid structures. A study by Kang et al. was among the first to report the presence of CD8^+^ T cells in ectopic lymphoid follicles in the joints of RA patients [40]. In addition, a recent study reported an increased frequency of CD8^+^CXCR5^+^PD1^+^ Tfh cells in LN tissues of IL-2 knock-out mice, which were shown to secrete IL-21 and promote B-cell antibody class switching. Interestingly, these CD8^+^ T follicular cells continued to expand in terms of frequency and numbers over time in these mice [20]. Although we did not investigate the B-cell class-switching potential of the observed lymph node CD8^+^ Tfh cells, their frequency correlated with the number of B cells. Human studies are needed to further unravel the potential of these cells to participate in a typical GC reaction, such as B-cell selection.

Since activated cells can leave the lymph node and travel towards the site of inflammation, we investigated whether inflamed synovial tissue harbors Tfh cells. We identified CXCR5^+^ICOS^+^Bcl-6^+^ Tfh cells in B-cell-rich areas of synovial tissue, where they may contribute to the formation of tertiary lymphoid structures and B-cell differentiation towards plasma cells, as described previously [41,42]. In line with our data, Tfh-like cells have been described to reside in inflamed synovium [43]. However, these Tfh cells were defined as CD4^+^CXCR5^+^ICOS^+^ cells without the identifying transcription factor Bcl6 [43]. Bcl-6 expression is absolutely required for Tfh cell differentiation and directs Tfh cell lineage commitment [44,45]. However, variable expression of Bcl6 in Tfh cells has been described, showing that when Tfh cells leave the GC, Bcl-6 expression is downregulated, but Bcl-6 expression is restored upon interaction with memory B cells [46]. Accordingly, we identified active Tfh cells based on their Bcl-6 expression and their presence in or near B-cell follicles of rheumatoid synovium. Although Tfh cells are thought to be crucial for B differentiation within GCs, a recent study identified another type of B-cell-promoting T helper cell in peripheral blood and synovial tissue expressing high levels of PD-1 but negative for CXCR5 [26]. These cells, identified as peripheral helper cells (Tph), express factors such as IL-21, CXCL13, and ICOS, enabling them to provide B-cell help and drive B-cell differentiation. Further characterization of Tph and Tfh cells in lymphoid tissues as well as inflamed synovial tissue is of interest to understand the mechanisms leading to the formation of secondary and tertiary lymphoid structures and subsequent autoimmune responses.

Similar to previous findings of decreased production of pro-inflammatory cytokines upon ex vivo stimulation of LN T cells of early RA patients [47,48], we found decreased production of IL-21 in LN Tfh cells of early RA patients and in some of the RA-risk individuals. This decreased production of IL-21 by CD4^+^CXCR5^+^ and CD8^+^CXCR5^+^ Tfh cells could be a consequence of prolonged antigen stimulation and exposure in vivo, resulting in an exhausted phenotype of these cells [49]. Interestingly, and in accordance with previous reports [47], this phenomenon of decreased IL-21 production by T cells in our study was only observed in LNs but not in peripheral blood. Our results further highlight the possibility that LNs may offer a unique environment that influences the function and phenotype of T cells resulting in disease development. Therefore, future work aimed at unraveling the possibility of follicular helper T cell exhaustion in lymphoid structures is needed. While IL-21 is described as the most important cytokine derived from Tfh cells to promote B-cell differentiation and proliferation, Tfh cells also produce other cytokines, such as IL-4 and IFN-gamma. Indeed, an in vitro study using sorted CD4^+^CXCR5^+^ blood Tfh cells from healthy controls (HCs) and chronic hepatitis C–infected patients (HCV) showed that Tfh cells from HCV patients produce less IL-21 but are equally capable of driving in vitro B-cell proliferation and differentiation into antibody-producing cells compared with healthy control cells [50]. This indicates that B-cell differentiation is not solely dependent on large amounts of IL-21. Another explanation for low ex vivo IL-21 production may be that primary immune responses are dependent on IL-21 but that secondary memory responses of Tfh cells are not [50,51].

Although the number of participants in our study is limited, the data presented here show for the first time the relation between the number of Tfh cells and B cells in human LN biopsies and increased frequencies of CD4^+^ and CD8^+^ Tfh cells in LN biopsies of early RA patients. Since Tfh may amplify B-cell responses and autoantibody production in lymphoid tissue and CD8^+^ Tfh cells are already increased in RA-risk individuals, targeting Tfh cells early could be tested as a new approach to prevent further disease progression during the earliest phases of RA.

## Figures and Tables

**Table 1:** Baseline characteristics of healthy controls (HCs), RA-risk individuals (RA-risk), and early RA patients (early RA).

	HC n = 17	RA-Risk n = 24	Early RA n = 16
Sex, female (%)	11 (65)	20 (83)	9 (56)
Age (years) ^#^	38.0 (15.91)	48.0 (12.9)	52.0 (14.1)
IgM-RF positive (n (%))	0 (0)	11 (46)	15 (94)
IgM-RF level (kU/L) *	3.3 (1.0–15.0)	21.0 (6.3–190.3)	312.0 (230.0–405.5)
ACPA positive (n (%))	0 (0)	15 (63)	15 (94)
ACPA level (kAU/L) *	4.0 (2.0–9.0)	45.0 (4.3–176.5)	388.0 (103.5–1529.5)
IgM-RF and ACPA both pos. (n (%))	0 (0)	2 (8)	14 (88)
ESR (mm/h) *	nd	7.5 (2.0–12.0)	13.5 (5.0–27.5)
CRP (mg/L) *	0.9 (0.5–2.3)	1.9 (0.9–3.8)	7.7 (4.5–13.2)
68 TJC (n) *	0 (0)	2.0 (1.0–3.8)	15.0 (9.8–21.8)
66 SJC (n) *	0 (0)	0 (0)	10.0 (5.0–13.3)

Categorical variables: n (%); continuous variables: #, mean with standard deviation (SD); *, median with interquartile range (IQR); ACPA, anticitrullinated protein antibodies; nd, not determined; ESR, erythrocyte sedimentation rate; CRP, C-reactive protein; IgM-RF, IgM rheumatoid factor; 68 TJC, tender joint count of 68 joints; 66 SJC, swollen joint count of 66 joints.

## Data Availability

All data relevant to the study are included in the article or uploaded as supplementary information.

## Supplementary Materials

The following supporting information can be downloaded online at: https://www.mdpi.com/article/10.3390/cells11071104/s1, Figure S1: Negative control (unstained) sample used for the setting of gates to analyze CXCR5, CCR7, PD-1, IL-21 and IL-10 cells in PBMCs.
