# Supplementary material for: Increased Frequency of CD4+ Follicular Helper T and CD8+ Follicular T Cells in Human Lymph Node Biopsies during the Earliest Stages of Rheumatoid Arthritis
Source: Cells. 2022 Mar 24;11(7):1104. doi: 10.3390/cells11071104 (PMC8997933; doi:10.3390/cells11071104)
Supplement: Supplementary file 1 [file cells-11-01104-s001.zip › cells-1600229-supplementary.pdf]

Supplemtary Figure S1

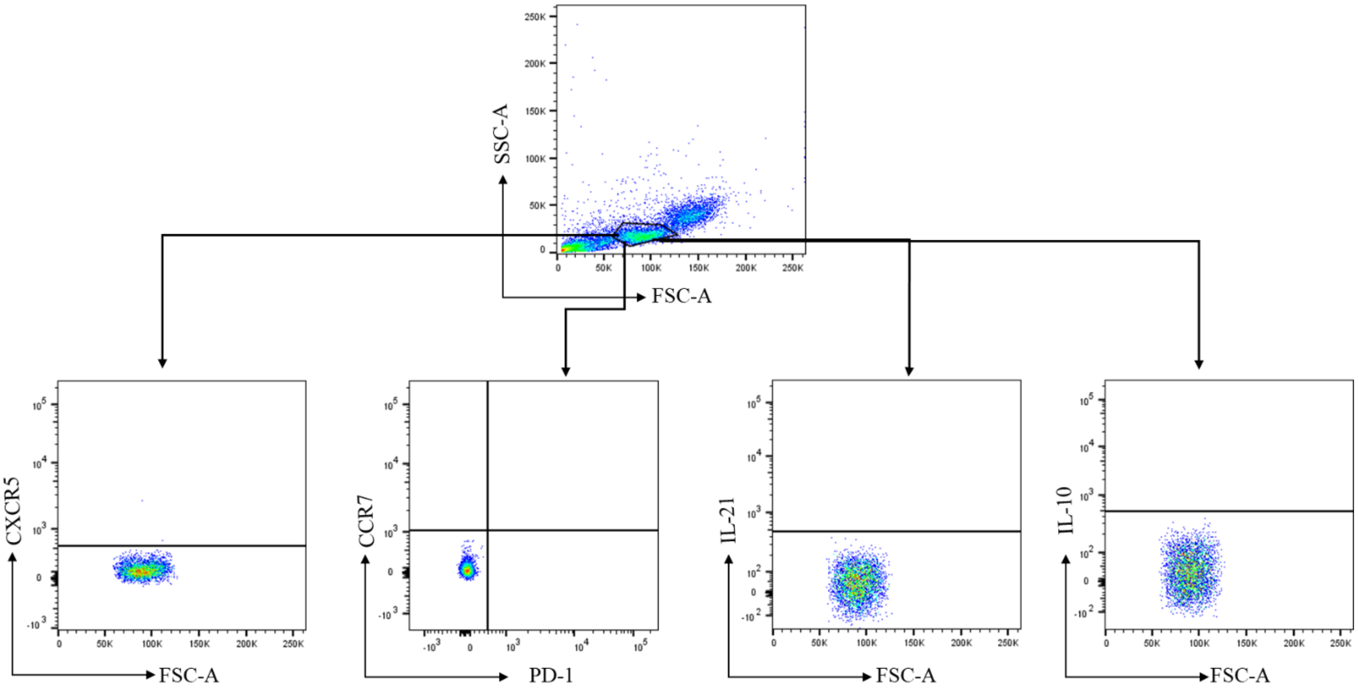

Supplementary figure S1 | Negatiev control (unstained sample used for the setting of gates to analyse CXCR5, CCR7, PD-1, IL-21 and IL-10 in PBMCs.
